# Comparison of immature and mature bone marrow-derived dendritic cells by atomic force microscopy

**DOI:** 10.1186/1556-276X-6-455

**Published:** 2011-07-16

**Authors:** Feiyue Xing, Jiongkun Wang, Mingqian Hu, Yu Yu, Guoliang Chen, Jing Liu

**Affiliations:** 1 Institute of Tissue Transplantation and Immunology, Jinan University, Guangzhou 510632, China; 2 Department of Chemistry, Jinan University, Guangzhou 510632, China; 3 Department of Immunology, H. Lee Moffitt Cancer Center and Research Institute, Tampa, FL 33612, USA; 4 Department of Blood and Marrow Transplantation, H. Lee Moffitt Cancer Center and Research Institute, Tampa, FL 33612, USA; 5 Department of Stomatology, Jinan University, Guangzhou 510632, China

**Keywords:** dendritic cell, nanostructure, adhesion force, comparison

## Abstract

A comparative study of immature and mature bone marrow-derived dendritic cells (BMDCs) was first performed through an atomic force microscope (AFM) to clarify differences of their nanostructure and adhesion force. AFM images revealed that the immature BMDCs treated by granulocyte macrophage-colony stimulating factor plus IL-4 mainly appeared round with smooth surface, whereas the mature BMDCs induced by lipopolysaccharide displayed an irregular shape with numerous pseudopodia or lamellapodia and ruffles on the cell membrane besides becoming larger, flatter, and longer. AFM quantitative analysis further showed that the surface roughness of the mature BMDCs greatly increased and that the adhesion force of them was fourfold more than that of the immature BMDCs. The nano-features of the mature BMDCs were supported by a high level of IL-12 produced from the mature BMDCs and high expression of MHC-II on the surface of them. These findings provide a new insight into the nanostructure of the immature and mature BMDCs.

## Introduction

Dendritic cells (DCs) are the most potent specialized antigen-presenting cells, which bridge the innate and adaptive immune response, controlling both immunity and tolerance. It is well known that DCs may be derived from bone marrow progenitors with two major developmental stages: immature and mature DCs [1]. The development of immature DCs can be induced with using cytokines, such as granulocyte macrophage-colony stimulating factor (GM-CSF) [2], FMS-like tyrosine kinase 3 (FLT3) [3], or cytokine cocktails containing GM-CSF +/-IL-4 [4] in vitro. After stimulation of lipopolysaccharide (LPS), poly I:C or thymic stromal lymphopoietin (TSLP), immature DCs can further differentiate into mature DCs, with increase of IL-12 and up-regulation of MHC-II, CD40, CD80, CD83, and CD86 molecules on the surface of DCs [5,6]. The maturation status of DCs is relatively important for them whether to induce immune tolerance or to initiate immune response. It is well proved that the transition from immature DCs to mature DCs is accompanied by morphological changes to be suitable for requirement of immunological function changes of DCs. Scanning electron microscopy (SEM) is a conventional tool for imaging cell morphology, which requires a conductive surface and a high-vacuum condition [7]. By contrast, atomic force microscopy (AFM), with continuously growing uses in investigating biomaterials, can be operated directly in air, vacuum, or physiological conditions with nanometer lateral resolution [7,8]. Furthermore, AFM is capable of providing quantitative analysis of cell surface and adhesion force features. Although the morphology of DCs has early been observed by conventional optical microcopy, SEM, and transmission electron microcopy methods [7,9], comparison of immature and mature DCs has not been, to date, carried out using AFM. Therefore, it is necessary to find out nanostructure of DCs, especially different nano-properties and adhesive force that cannot be discovered by optical and electron microscopy. In this study, AFM was exploited to reveal differences of the nano-features and adhesive force between both immature and mature bone marrow-derived dendritic cells (BMDCs). Obviously, this study would provide a novel insight into the nanostructure and force feature of immature and mature DCs.

## Materials and methods

### Preparation of bone marrow cells

Bone marrow-derived dendritic cells were generated according to Lutz's publication [10] with a little modification. In brief, cervical cords in female Balb/c mice with 6 to 8 weeks old (Sun Yat-sen University, Guangzhou, China) were mechanically dislocated to sacrifice them. After removing all muscle tissues from the femurs and tibias, intact bones were left in 70% ethanol for 2 to 5 min for disinfection and washed with phosphate-buffered saline (PBS). Then, both ends were cut with scissors and the marrow was washed with PBS through a syringe. Clusters within the marrow suspension were disintegrated by vigorous pipetting. The bone marrow cell suspension was centrifuged at 300 × *g *for 5 min. The cells were collected, suspended in PBS by addition of red blood cell lysate for depletion of erythrocytes, and incubated at 37.0°C for 8 min away from light. Then, they were washed with PBS at 300 × *g *for 5 min three times. At last, the cells were harvested and resuspended in RPMI1640 (Gibco BRL, Gaithersburg, MD, USA) complete culture medium containing 10% (*v*/*v*) fetal bovine serum (FBS) (Gibco BRL), 2 mmol/L L-glutamine, 10 μmol/L 2-mercaptoethanol (Sigma-Aldrich, St Louis, MO, USA), 100 U/mL penicillin and 100 μg/mL streptomycin, and adjusted to 2 × 10^9^/L.

### Induction and separation of bone marrow-derived dendritic cells

The above cells were seeded into a 6-well plate to the end volume of 2 mL per well, and 10.0 μg/L of rmGM-CSF (PeproTech, Rocky Hill, NJ, USA) plus 10.0 μg/L of rmIL-4 (PeproTech) was added to the corresponding wells in the plate and cultured at 37.0°C in an incubator containing 5% CO_2 _to induce differentiation of bone marrow cells into bone marrow-derived dendritic cells. Then, the cells were fed once at the interval of 1 day with the identical dose of rmGM-CSF plus rmIL-4 for 6 days. At the end of the cell induction, all the cells expressing CD11c in the different wells were isolated respectively using the Mouse CD11c Positive Selection Kit (EasySep^®^Magnet, StemCell Technologies, Vancouver, Canada) according to the manufacturer's instruction and seeded into new wells with fresh medium. Finally, the CD11c-positive cells were treated with or without LPS (Sigma-Aldrich) at a dose of 1.0 mg/L for another 24 h in order to obtain mature BMDCs.

### Scanning electron microscopy

After the stimulation of LPS, the CD11c-positive cells were rinsed with PBS containing 0.5 mM MgCl2 and 1 mM CaCl2, made naturally subside to the glutin-coated glass for 10 min, then fixed at 4°C for 30 min with 2.5% glutaraldehyde in 0.1 M phosphate buffer, pH 7.4, and post-fixed for 30 min with 1% osmium tetroxide in 0.1 M phosphate buffer, pH 7.4. The glass was gradually dehydrated in ethanol (30%, 50%, 70%, 90%, and twice in 100% for 5 min at each step) and subjected to critical point drying using carbon dioxide as transitional medium. The samples were stored in a vacuum exsiccator to prevent putative deterioration by air humidity. Then, they were connected to stub holders with liquid silver paint to improve electrical conductivity and imaged in SEM (ESEM-30, Philips, Mahwah, NJ, USA) with a field emission electron gun operating at standard high-vacuum settings.

### Flow cytometry

The CD11c-positive cells were harvested after the selection of immunomagnetic beads and the stimulation of LPS as described above. After being centrifuged, they were washed with PBS at 300 × *g *for 5 min and resuspended in PBS. Then, the cells were stained with both 0.25 μg anti-CD11c-FITC and 1.0 μg anti-MHC-II-PE (eBioscience, USA) per million cells in a 100μl total volume. After being mixed gently on a vortex machine, they were placed at 4.0°C in the dark for 30 min, and then rinsed with PBS for two times and centrifuged at 300 × *g *for 5 min. The expression level of CD11c on the surface of the cells was analyzed by flow cytometry (FAC-Scalibur, Becton Dickinson, Franklin Lakes, NJ, USA). A total of 5 × 10^3 ^events were analyzed for each determination and calculated by CellQuest software (Becton Dickinson).

### ELISA

The above selected BMDCs were treated with or without LPS at a concentration of 1.0 mg/L for 24 h. Their culture supernatant was collected. The level of IL-12 in the supernatant was determined via enzyme linked immunosorbent assay (ELISA) with the IL-12 ELISA Kit (Bender MedSystems, Burlingame, CA, USA) according to the manufacturer's protocol. Absorbance value was measured at 450 nm in 680 type microplate reader (Bio-Rad, Berkeley, CA, USA). The concentration of IL-12 was quantified according to a standard curve.

### AFM analysis

AFM observation was performed according to the reported method [11,12]. In brief, the mica carrying the BMDCs was fixed for 15 min in 2% glutaraldehyde phosphate buffer at pH 7.4, washed gently with distilled water three times, and dried naturally. Then, contact mode scanning was immediately performed using a commercial AFM (AutoProbe CP Research, Thermomicroscopes, Sunnyvale, CA, USA) in air at room temperature. The curvature radius of the silicon nitride tip (UL20B, Park Scientific Instruments) was around 10 nm, and a force constant about 2.8 N/m was used. To obtain high resolution, we scanned samples at rate of 0.3 Hz. All of the AFM images were flattened with provided software (Thermomicroscopes Proscan Image Processing Software Version 2.1) to complete quantitative analysis.

An autoprobe CP AFM was used in a contact mode in air to perform the topography images at room temperature according to the publications [11-14]. AFM-based force spectroscopy was used to perform the force detection. The same silicon nitride tip was applied for measurement of all the force-distance curves at the same speed. Force-distance curves were obtained through standard retraction between the tip and cell surface. Two hundred fifty-six force-distance curves were recorded for every cell (*n *= 10 cells for each group). All force-distance curve experiments were performed at the same loading rate.

The root-mean-square (rms) roughness and average roughness of the cell surface imaged in air were calculated using the AFM. The rms roughness (*R*_rms _or *R*_q_) and average roughness (*R*_a_) were defined by formulas below:

where *N *is a total quantity of measured spots, *Z*_n _means a height of any spot, and  represents an average height of all the spots. The calculated *R*_rms _and *R*_a _refer only to the area shown in the top central part of the cells.

### Statistical analysis

Numerical data obtained from each experiment were expressed as mean ± SD, analyzed by SPSS 10.0 statistical package. The Student's *t *test was followed for data comparison and a *P *value of less than 0.05 was considered statistically significant.

## Results and discussion

### Morphologic and functional characteristics of BMDCs

**Figure 1:**
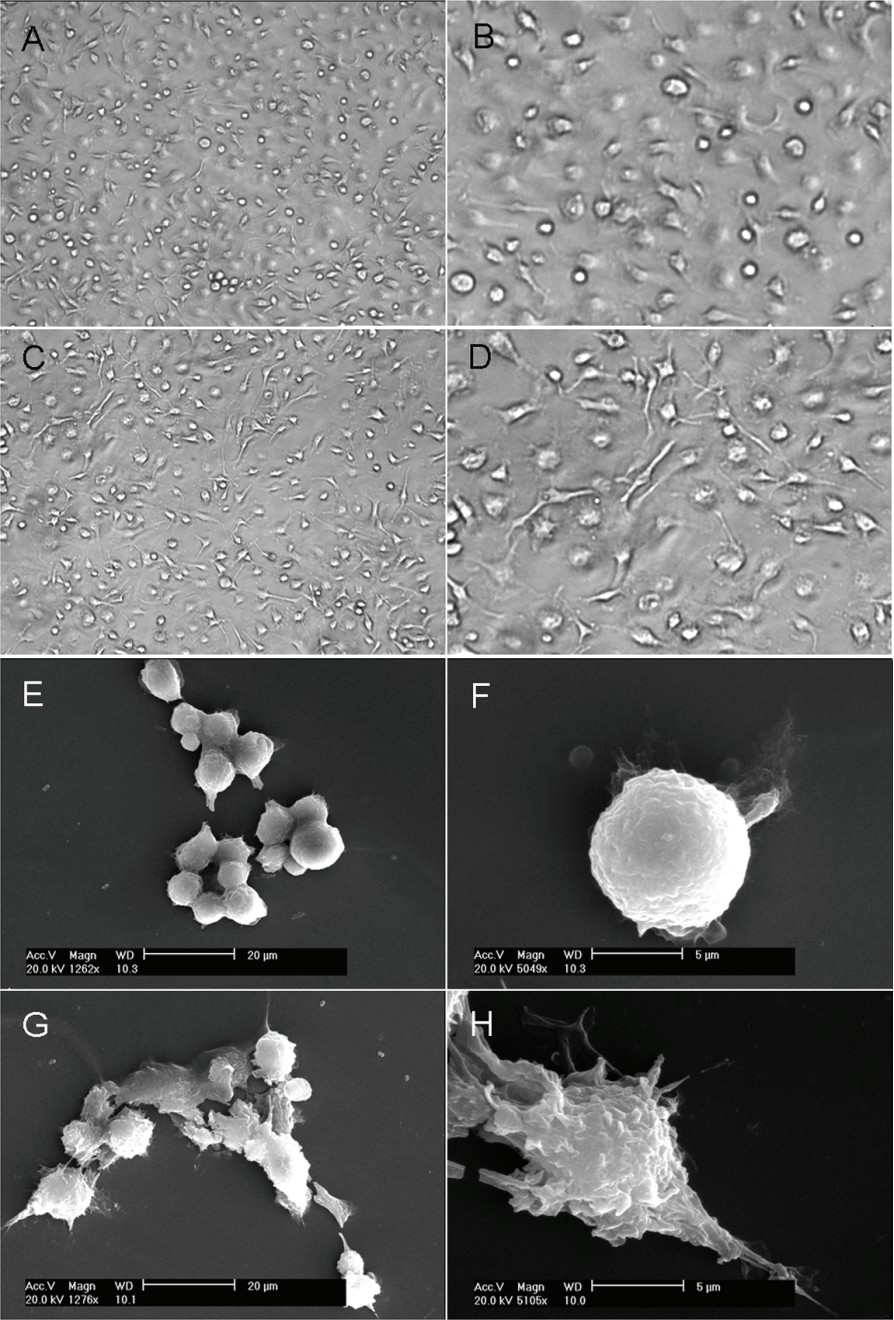
**Morphologic changes of immature and mature BMDCs**. (A, B) The morphology of the BMDCs treated with GM-CSF plus IL-4 was observed under a light microscope (magnification: ×100 (A) and ×400 (B)). (C, D) The morphology of the BMDCs stimulated with LPS was also done under a light microscope (magnification: × 100 (C) and × 400 (D)). (E, F) The images of the BMDCs treated with GM-CSF plus IL-4 were scanned by a scanning electron microscope (SEM) with different magnifications, including around ×1,200 (E) and ×5,000 (F); (G, H) SEM images of the BMDCs stimulated with LPS were recorded with different magnifications, i.e., around ×1,200 (G) and ×5,000 (H).

**Figure 2:**
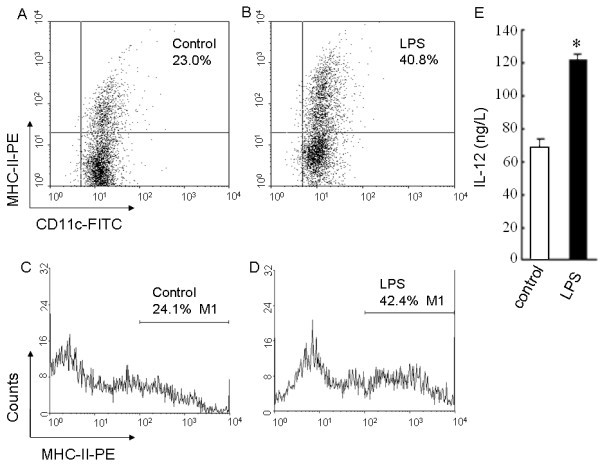
**MHC-II expression and IL-12 production of immature and mature BMDCs**. (A-D) Flow cytometry was used to detect CD11c and MHC-II molecule expression on the surface of the immature BMDCs treated with 10.0 μg/L of rmGM-CSF plus 10.0 μg/L of rmIL-4 as the control (A, C) or the mature BMDCs stimulated with 1.0 mg/L of LPS (B, D), which was displayed respectively by the scattered plots (A, B) and the single parameter diagrams (C, D). (E) The level of IL-12 secreted by the immature BMDCs or the mature BMDCs was measured by ELISA. *P < 0.05, compared with the control.

The bone marrow cells were cultured and induced in complete RPMI 1640 medium supplemented with a given dose of GM-CSF plus IL-4 for 6 days. Six days post induction of rmGM-CSF plus rmIL-4, the BMDCs appeared predominately round in loosely adhesive growth under a light microscope (Figure 1A,B) and SEM (Figure 1E,F). When observed at a high resolution, the BMDCs were ridgy in shape with a relative smooth membrane surface (Figure 1F), demonstrating that they are mostly in immature status. But the BMDCs with treatment of LPS (LPS-treated BMDCs) changed greatly under a light microscope (Figure 1C,D) and SEM (Figure 1G,H). After treatment of LPS, some of BMDC became significantly larger in size with rough surface, richer ruffles on the cell membrane, and bigger, longer protrusions or pseudopodia (Figure 1G,H), compared with the control (Figure 1E,F). The formation of roughness, protrusion, and ruffles on the cell membrane are considered to be associated with maturation of BMDCs. These results suggest that there exist obviously morphologic characteristics of mature BMDCs, consistent with previously reported data [15]. Generally speaking, it is considered that the morphologic change is the foundation of the phenotype and the function of BMDCs. MHC-II is one of activation molecules expressed on the surface of BMDCs, representing a phenotype of mature BMDCs. Flow cytometry analysis showed that the percentage of CD11c^+^MHC-II^+ ^cells in LPS-treated BMDCs was twofold more than that in BMDCs (Figure 2A,D), indicating that some of the LPS-stimulated BMDCs become mature. This is supported by our finding that the percentages of CD11c^+^CD86^+ ^cells, CD11c^+^CD80^+ ^cells, and CD11c^+^CD40^+ ^cells in LPS-treated BMDCs were 1.5-, 1.6-, and 2.5-fold more than those in BMDCs, respectively [16]. IL-12 release is a functional characteristic of DC maturation and also crucial for mature DCs to mediate Th1 differentiation so as to enhance immune responses. Mature DCs can direct differentiation of naïve CD4^+ ^T cells into Th1 cells through IL-12 and interaction between DCs and the latter [17-19]. Therefore, we further examined whether BMDCs treated with LPS were of a functional feature of DC maturation. The amount of IL-12 in culture supernatants of BMDCs was assessed by ELISA. Compared with the control, LPS promoted significantly secretion of IL-12 by BMDCs (Figure 2E). In terms of previous reports, nuclear factor (NF)-kappaB plays a major role in regulation of DC maturation, and LPS-mediated activation of NF-kappaB in DCs leads to the production of IL-12 [20,21]. These results suggest that BMDCs acquire maturation after treatment of LPS, consistent with up-regulation of a co-stimulating molecule, MHC-II, on the surface of DCs. The forgoing findings from morphology, phenotype, and function of BMDCs indicate that there are distinct differences between both the immature and mature BMDCs. The confirmed immature and mature BMDCs have been successfully induced, isolated, and identified, being suitable further for a comparative study by AFM.

### Nano-structural comparison of immature and mature BMDCs

**Figure 3:**
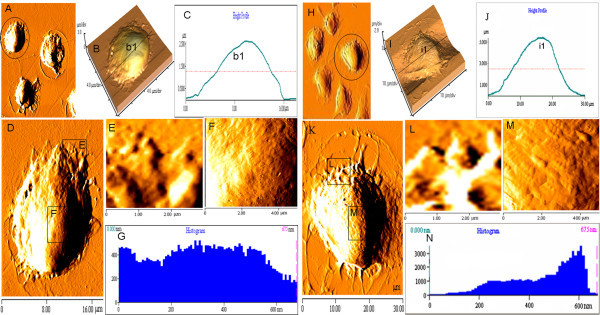
**Nanostructure on the surface of immature and mature BMDCs**. (A-N) AFM was adopted to determine nanostructures of the immature BMDCs treated with 10.0 μg/L of GM-CSF plus 10.0 μg/L of IL-4 (A-G) or the mature BMDCs stimulated with 1.0 mg/L of LPS (H-N), and to make quantitatively analysis for them; A and H, multiple immature BMDCs (A) or mature BMDCs (H) at lower resolution; B and I, three-dimensional images respectively from the black line-circled cells on A and H images; C and J, height profiles alone the black lines (b1 and i1) drawn across the cells on B and I images, respectively; D and K, single immature BMDC in scanning area of 18 × 18 μm (D) or single mature BMDC in scanning area of 30 × 30 μm (K) respectively from the black line-circled cells on A and H images; E and L, Enlarged view of the protrusion or pseudopodia on the edge of the immature BMDCs (E) in the scanning size of 3 × 3 μm and the mature BMDCs (L) in the scanning size of 3 × 3 μm; F and M, Enlarged view of the center of the immature BMDCs (F) and the mature BMDCs (M) in the same scanning area of 5 × 5 μm; G and N, histograms of the particles of the immature BMDCs (G) and the mature BMDCs (N).

**Figure 4:**
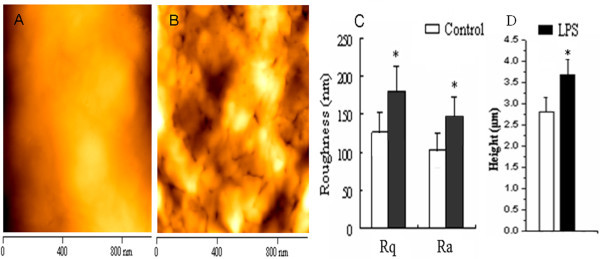
**Quantitative analysis of the surface roughness and the height of immature and mature BMDCs**. (A, B) AFM was exploited to show topographic images of the surface nanostructure of the immature BMDCs treated with 10.0 μg/L of GM-CSF plus 10.0 μg/L of IL-4 (A) as the control or the mature BMDCs stimulated with 1.0 mg/L of LPS (B) in the same scanning area of 5 × 5 μm; (C) The root-mean-square roughness (*R*_rms _or *R*_q_) and average roughness (*R*_a_) on the surface of the immature BMDCs (A) and the mature BMDCs (B) were quantitatively analyzed via the formulas as described in the section of "Materials and methods." (D) The average heights of immature and mature BMDCs were statistically quantified, respectively. *n *= 10; *P < 0.05, compared with the control.

Compared with both optical microscopy and SEM, AFM has some unique advantages, such as clearer images, easy sample preparation, extensive environments (in air or liquid allowing cells to "stay alive") of sample to escape from the damage of reagents, strong electrical field, and ultrahigh vacuum in electron microscopy, and so on [22,23]. Therefore, a comparative study of immature and immature BMDCs was carried out by AFM to visualize and quantify nanostructures of them. AFM images included single and multiple BMDCs, two and three dimensions, low and high resolutions, cell height profile and histogram, topography, and roughness on the surface of the cells (Figure 3). The immature BMDCs treated with rmGM-CSF plus rmIL-4 were shown on Figure 3A-3G, and the mature BMDCs stimulated by addition of LPS on Figure 3H-3N, which provided the quantitative topographic information and the error signal images for revealing fine surface details. The immature BMDCs appeared mainly round, and around 18 × 18 μm in scanning area (Figure 3B,D) with uniformly smooth cell surface and approximately 2.5 μm in height on the center (Figure 3C). However, the mature BMDCs displayed an irregular shape with numerous pseudopodia or lamellapodia, and ridgy and ruffles on the surface of the cell membrane in addition to becoming larger and longer. Some of them were around 30 × 30 μm in scanning area (Figure 3I,K) and approximately 5.0 μm in height on the center (Figure 3J); 5 × 5 μm of the area was scanned respectively on the edge and top surface of the cells (Figure 3E,F,L,M). Quantitative analysis showed that the granule size on the surface of the mature BMDCs (Figure 3M,N) was much higher than that of the immature BMDCs (Figure 3F,G). At the edge of the mature BMDCs, there were some longer and more pseudopods (Figure 3K,L), but shorter and less ones in the immature BMDCs could be found (Figure 3D,E). The roughness on the surface of the mature BMDCs (Figure 3M,N) was much higher than that of the immature BMDCs as well (Figure 3F,G and Figure 4). There exist, to date, no detailed reports involving nanostructure comparison of both immature and mature BMDCs. Thus, the foregoing results would be helpful for profoundly understanding the morphologic properties and functional foundation of both immature and mature BMDCs. Obviously, AFM-revealed features could not be replaced by SEM. The difference between the spatial resolutions may be due to different principles exploited by both SEM and AFM. AFM scans cell surface with a tip probe, whereas SEM uses an electron beam to obtain the image of cell surface [7]. Besides, easy sample preparation without conductive coating could protect AFM image from damage of the sample [22,24]. In addition to providing topographical images of cell surfaces with nanometer- to angstrom-scale resolution, forces between single molecule and mechanical property of cells can be investigated by AFM. This quality can distinguish AFM from conventional imaging techniques of comparable resolution, such as electron microscopy, too.

Regarding the enhancement of the mature DC height and volume, it is associated with the differentiation and maturation of DCs induced by LPS. It is well known that LPS can activate Toll-like receptor 4 on the surface of immature DCs. The activation of a Toll-like receptor 4 signaling pathway finally causes nuclear translocation of the nuclear factor (NF)-kappaB transcription factor. The inhibition of NF-kappaB activation blocks maturation of DCs, followed by down-regulation of major histocompatibility complex and co-stimulatory molecules, which indicates that the activated NF-kappaB signaling pathway may be responsible for DC maturation. Simultaneously, it is found that LPS activates the extracellular signal-regulated kinase1/2 (ERK1/2) in DCs. The specific inhibition of MEK1, an upstream kinase of ERK1/2, abrogates the ability of LPS to prevent apoptosis but does not impact the DC maturation, which suggests that ERK1/2 signaling pathway may mainly maintain DC survival [25]. Ardeshna's research group showed that LPS activated the p38 mitogen-activated protein kinase (p38 MAPK), ERK1/2, phosphoinositide 3-OH kinase (PI3 kinase)/Akt, and NF-kappaB pathways in the process of DC maturation. PI3 kinase/Akt signaling pathways are important in maintaining survival of LPS-stimulated DCs. Inhibiting p38 MAPK prevented activation of the transcription factor ATF-2 and CREB, and significantly reduced the LPS-induced up-regulation of co-stimulatory molecules [26]. It is also demonstrated by another research group's results that ERK1/2, p38MAPK, c-jun N-terminal kinase (JNK), and NF-kappaB signaling pathways are implicated in the events of DCs maturation [27]. The differentiation and maturation of DCs require more synthetic materials and energy production, with enhancement of the whole cellular or subcellular metabolism and function. Morphological changes of cells are foundation of their metabolism and function changes, adapting to the need of the both latters. The big increase of subcellular organelles in LPS-stimulated mature DCs, especially including lysosome, mitochondrium, and endoplasmic reticulum with enrichment of cytoplasm, can be observed under a transmitted electronic microscope, finally resulting in the augmentation of the DC height and volume. The increase of mature DC surface area may be helpful for the expression of co-stimulatory molecules and relevant receptors on the surface of mature DCs, promoting intercellular interaction of mature DCs and other associated cells. Of course, these morphological changes of mature DCs may be regulated by the foregoing different and sometimes overlapping pathways.

### Adhesive force comparison of immature and mature BMDCs

**Figure 5:**
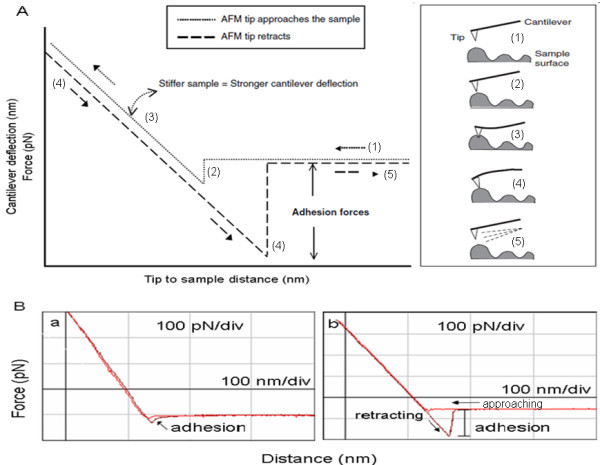
**Adhesive force of immature and mature BMDCs**. (A) As shown in Figure 5A (slightly modified from Shahin et al.[29,30]), the AFM tip is moved toward the cell surface (1) and then retracted at a constant lateral position (2) and (3). During the AFM tip retraction, the AFM tip with the sample leads to a force signal with a distinct shape (4). The force increases until bond rupture occurs (5) at an unbinding force; (B a and b) The typical force-distance curves were recorded with using an non-functionalized AFM tip to measure the adhesive force of the immature BMDCs treated with 10.0 μg/L of GM-CSF plus 10.0 μg/L of IL-4 (B a) or the mature BMDCs stimulated with 1.0 mg/L of LPS (B b). The measured adhesion force (352.37 ± 11.71 pN) on the membrane surface of the mature BMDCs was much bigger than that (70.37 ± 4.55 pN) of the immature BMDCs (*n *= 10; *P *< 0.01).

Operational principle of AFM was schematically shown in Figure 5A. Schematic representation of a typical force-distance cycle was used to display the full process of measuring cell adhesion force. The tip was moved toward the cell surface (1) and (2), and then retracted at a constant lateral position (3). During tip approach, the tip with the sample leaded to a force signal with a distinct shape (4) during tip retraction. The force increased until bond rupture occurred (5) at an unbinding force [28-30]. Two force-distance curves recorded between the silicon nitride probe and the surface of the BMDCs were shown in Figure 5. Force-distance curve measurement demonstrated that the changes in the immature or mature BDMC surface nanostructure went along with profound modification of the nanomechanical property. Upon approach, no significant deviation from linearity was seen in the contact region of the immature BDMCs, indicating that the sample was not deformed by the probe. Upon retraction, the adhesion force was detected, reflecting the absence of molecular interaction between both probe and surface. In contrast with the immature BDMCs, the mature BDMCs revealed a curvature upon approach, reflecting sample softness and/or repulsive surface forces. This might be due to electrostatic interaction. Furthermore, silicon nitride surface was shown to be close to electrical neutrality over a wide pH range (pH 6 to 8.5). The heterogeneous surface of BDMCs after addition of GM-CSF or LPS was directly correlated with differences in adhesion force revealed by retraction curves. The weak adhesion force was measured between the probe and the immature BDMC surface, being around 50 to 80 pN (Figure 5B a), while great adhesion force was determined on the mature BDMCs, being fourfold bigger than the former (*n *= 10 cells for each group) (Figure 5B b). All of the 256 force-distance curves recorded showed the same feature, indicating that the sample surface was homogeneous as regards the nanomechanical property. It has been proved that polysaccharides play a key role in cellular adhesion [31]. Thus, the increased adhesion force on the surface of the mature BDMCs might be attributed to the presence of polysaccharide aggregation and mechanically beneficial to deformation, movement, migration, adhesion, and interaction of the mature BMDCs, which may adapt to functional changes of them. In addition, it should be pointed out that adhesive force-distance curve measurement was processed only using fixed BMDCs due to the limitation of the used instrument. Therefore, it is reasonably speculated that the measured adhesive force might be smaller than that under the physiological state of living BMDCs. Obviously, BMDCs growing in culture medium merit to be directly observed to explore it using a more advanced AFM. Moreover, antigen-antibody interaction force on the surface of mature BMDCs remains investigated further by using chemically modified probes. This would provide a new insight into molecular mechanisms of bio-interfacial phenomena, including aggregation, adhesion, molecular recognition, and intercellular communication of the mature BMDCs.

It should be pointed out that the AFM tip is going to be contaminated at the first touch and continue with the following touches, and this contamination can influence the next interaction of the tip with the cells. Therefore, contamination control of AFM tips is very important for reliable AFM imaging and surface/interface force measurements. Most contaminants may result in poor imaging quality either by causing tip effects and/or noise [32]. Tip effects reflect the increase in tip size as the contaminants add to the tip apex [33]. A noisy AFM image can be a result of uncontrollable interaction (such as sudden bridging or breaking) between the tip and the sample surface mediated by interspersed sticky contaminants. Nie et al. considered that such a contaminant confined on the tip apex displays an uncontrollable variation in the oscillation amplitude of the cantilever, causing noise in the AFM images the contaminated tip collects, but such a contaminant may be removed from the apex by pushing the tip into a material soft enough to avoid damage to the tip [34]. According to our experience, cell samples should be gently washed with the buffer at least three times for removing debris attachment from cell culture media and themselves before AFM determination. We think that a contact mode for the determination may be replaced by a tapping mode in order to reduce the contamination and cell damage if serious contamination occurs. Actually, traditional cleaning methods for the tip, including plasma, UV-ozone, solvent treatments, and so on, have been abroad applied, but there still are some shortcomings. Recently, Gan et al. reported that calibration gratings with supersharp spikes could be employed to scrub away contaminants accumulated on a colloidal sphere probe by scanning the probe against the spikes at high load at constant-force mode. This method may be superior to traditional cleaning methods in several aspects [35]. Anyway, control of AFM tip contamination is an extremely common issue and remains to be further studied.

Taken together, the above results first reveal the characterization of the surface nanostructure and adhesion force of the immature and mature BMDCs, providing profoundly understanding structure/function relationship of BMDCs.

## Competing interests

The authors declare that they have no competing interests.

## Authors' contributions

JW carried out the experiment, statistical analysis and participated in the draft of the manuscript. MH carried out AFM analysis. YY offered the technique supports. GC participated in the cell culture. JL conceived of the study, participated in the designs and was responsible for the experimental coordination. FX designed and participated in the experiment, drafted the manuscript, and was responsible for its coordination. All authors read and approved the final manuscript.
